# Exclusion of the genes *CDKN2* and *PTEN* as causative gene defects in Li–Fraumeni syndrome

**DOI:** 10.1038/sj.bjc.6690313

**Published:** 1999-04

**Authors:** E C Burt, G McGown, M Thorncroft, L A James, J M Birch, J M Varley

**Affiliations:** CRC Section of Molecular Genetics, Paterson Institute for Cancer Research, Wilmslow Road, Manchester M20 4BX, UK; CRC Paediatric and Familial Cancer Research Group, Royal Manchester Children's Hospital, Manchester M27 1HA, UK

**Keywords:** Li–Fraumeni syndrome, *PTEN*, *CDKN2*, p16, p19^ARF^

## Abstract

We have analysed Li–Fraumeni syndrome families, previously shown to be negative for mutations in *TP53*, for mutations to the tumour suppressor genes *PTEN* and *CDKN2*. These genes function in cell cycle progression or are mutated in a variety of tumours. We have detected no mutations in the family members tested. © 1999 Cancer Research Campaign


British Journal of Cancer (1999) 80(1/2), 9–10
© 1999 Cancer Research Campaign
Article no. bjoc.1998.0313

### Exclusion of the genes *CDKN2* and *PTEN* as causative gene defects in Li–Fraumeni syndrome

**EC Burt[1], G McGown[1], M Thorncroft[1], LA James[1], JM Birch[2] and JM Varley[1]**

[1]CRC Section of Molecular Genetics, Paterson Institute for Cancer Research, Wilmslow Road, Manchester M20 4BX, UK; [2]CRC Paediatric and Familial Cancer Research Group, Royal Manchester Children's Hospital, Manchester M27 1HA, UK

**Summary** We have analysed Li–Fraumeni syndrome families, previously shown to be negative for mutations in *TP53*, for mutations to the tumour suppressor genes *PTEN* and *CDKN2*. These genes function in cell cycle progression or are mutated in a variety of tumours. We have detected no mutations in the family members tested.

**Keywords:** Li–Fraumeni syndrome; *PTEN*; *CDKN2*; p16; $p19^{ARF}$

Li–Fraumeni syndrome (LFS) is an autosomal, dominant, inherited cancer disorder, characterized by a broad but specific spectrum of tumours observed at an early age, in particular a predominance of bone and soft tissue sarcomas, brain and breast cancer (Li et al, 1988). LFS is defined by a strict clinical definition, described previously (Li et al, 1988). This definition has been relaxed to include Li–Fraumeni-like (LFL) cases (Birch et al, 1994).

In 1990, the tumour suppressor gene *TP53* was identified as the candidate for mutation in a number of LFS families (Malkin et al, 1990). To date more than 50 families have been identified with germline *TP53* mutations (Varley et al, 1997*a*). Previously we have detected mutations in approximately 70% of LFS and 20% of LFL families, when all exons, including non-coding regions, have been sequenced (Varley et al, 1997*b*). Therefore, 30% of LFS and the majority of LFL families do not have a detectable germline mutation within the coding region of *TP53*. The p53 protein functions in checkpoint control at G1/S and G2 phase of the cell cycle. It is feasible that characterization of related genes, for example other cell cycle inhibitors, may identify germline mutations within these families. We have selected genes for analysis that are involved in p53-related pathways or are known to be mutated in Li–Fraumeni-associated tumours, in particular the tumour suppressor genes *CDKN2*, which encodes p16 and $p19^{ARF}$, and *PTEN*. Like p53, both p16 and $p19^{ARF}$ function as negative regulators of the cell cycle, with the p16 gene shown to be mutated in many tumour types (Kamb, 1995; Quelle et al, 1995). *PTEN* mutations have also been observed in a number of tumours (Steck et al, 1997). We have analysed 16 families, previously shown to be negative for mutations to *TP53* (Varley et al, 1997*b*), for mutations to these sequences.

*Received 7 August 1998*
*Revised 28 October 1998*
*Accepted 29 October 1998*

*Correspondence to:* JM Varley

### MATERIALS AND METHODS

Families were ascertained as previously described (Varley et al, 1997*b*). Blood samples were obtained from either the proband or a first-degree relative in each family (Table 1). DNA was isolated from whole blood using the Dynabeads DNA direct System (Dynal), according to manufacturers instructions.

Genomic DNAs were amplified by nested polymerase chain reaction (PCR) to generate products corresponding to each exon of *PTEN* and *CDKN2*. Primers and PCR conditions are available from authors. PCR products were sequenced directly using either an ABI377 Sequencer with Dye Primer chemistry (Perkin-Elmer Applied Biosystems), or a Sequenase kit (USB).

**Table 1** Mutation analysis of *PTEN* and *CDKN2* in LFS and LFL families[a]

| Family[b] | Person[b] | Mutation found[c] | |
|---|---|---|---|
| | | Nucleotide change[d] | Amino acid change |
| LFS Families | | | |
| 22 | III-5 | None | |
| 81 | III-5 | None | |
| 82 | IV-5 | None | |
| 88 | II-2 | None | |
| 119 | III-1 | None | |
| 1779 | III-7 | None | |
| LFL Families | | | |
| 80 | V-4 | None | |
| 253 | IV-2 | *CDKN2* : 442 G>A | p16: Alal48Thr[e] |
| 328 | II-3 | None | |
| 338 | III-2 | None | |
| 348 | III-2 | None | |
| 352 | IV-1 | None | |
| 353 | III-3 | None | |
| 2093 | IV-3 | None | |
| 2613 | III-1 | None | |
| 2634 | III-3 | None | |

[a]For details of tumour types and relationship to proband in each family, refer to Varley et al (1997*b*). [b]Family and Person numbers are as previously (Varley et al, 1997*b*). [c]Includes mutations found in *PTEN* and *CDKN2*. [d]Nucleotide positions are derived from the amended p16 gene sequence (Okamoto et al, 1994). [e]Ala148Thr is a common polymorphism, previously reported as Ala140Thr (Hussussian et al, 1994).

### RESULTS AND DISCUSSION

We report the results of sequencing six LFS and ten LFL samples for mutations in tumour suppressor genes *PTEN* and *CDKN2*. The *CDKN2* gene consists of four exons (1α, 1β, 2 and 3), and encodes two proteins: p16 and p19$^{ARF}$. The p16 protein is encoded by exons 1α, 2 and 3, and functions as a negative regulator in the G1 phase of the cell cycle. The *p16* gene is a major target in carcinogenesis, and has been shown to be inactivated in a number of tumour cell lines and primary tumours including adenocarcinomas and glioblastoma (Kamb, 1995). Germline mutations in the gene can result in hereditary predisposition to the development of melanoma and pancreatic cancer (Hussussian et al, 1994; Foulkes et al, 1997). In addition, an alternatively spliced transcript, encoding p19$^{ARF}$, is derived from the same locus as p16 and contains a novel first exon (1β), located upstream of exon 1α. Both transcripts share exons 2 and 3 but are translated in a different reading frame, and hence p16 and p19$^{ARF}$ have no amino acid similarity. The p19$^{ARF}$ protein also functions to block cell cycle progression at G1 and G2, although in a p53-dependent manner (Quelle et al, 1995). We have been unable to identify any germline mutations in either the *p16* or *p19$^{ARF}$* genes in the Li–Fraumeni families tested, although we have detected a single heterozygous polymorphism at codon 148 of p16 in IV-2, family 253 (Table 1). This polymorphism is prevalent in the population (Hussussian et al, 1994), and has no effect on p19$^{ARF}$.

The tumour suppressor gene *PTEN* (*MMAC1*) was isolated by mapping of homozygous deletions commonly found in glioblastomas (Li et al, 1997). Subsequently somatic mutations in *PTEN* have been observed in a number of tumours including breast and brain tumours, and malignant melanoma (Steck et al, 1997). Germline mutations in the gene have been identified in Cowden syndrome, which is associated with early onset breast cancer (Tsou et al, 1997). However, a recent study of 136 breast cancer families has shown that *PTEN* is not linked to familial breast cancer (Chen et al, 1998). The gene encodes a dual specificity phosphatase, with enzymatic activity required for tumour suppressor function (Myers et al, 1997). We have not detected any *PTEN* mutations in the families analysed.

We have therefore excluded these genes as candidates for mutation in Li–Fraumeni syndrome. However, this does not preclude other cell cycle control genes as targets for mutation, for example the Rb and p21 genes.

### REFERENCES

Birch JM, Hartley AL, Tricker KJ, Prosser J, Condie A, Kelsey AM, Harris M, Morris Jones PH, Binchy A, Crowther D, Craft AW, Eden OB, Evans DGR, Thompson E, Mann JR, Martin J, Mitchell ELD and Santibanez-Koref MF (1994) Prevalence and diversity of constitutional mutations in the *p53* gene among 21 Li–Fraumeni families. *Cancer Res* **54**: 1298–1304

Chen J, Lindblom P and Lindblom A (1998) A study of the *PTEN/MMAC1* gene in 136 breast cancer families. *Hum Genet* **102**: 124–125

Foulkes WD, Flanders TY, Pollock PM and Hayward NK (1997) The *CDKN2A* (*p16*) gene and human cancer. *Mol Med* **3**: 5–20

Hussussian CJ, Struewing JP, Goldstein AM, Higgins PAT, Ally DS, Sheahan MD, Clark WH Jr, Tucker MA and Dracopoli NC (1994) Germline p16 mutations in familial melanoma. *Nature Genet* **8**: 15–21

Kamb A (1995) Cell-cycle regulators and cancer. *TIG* **11**: 136–140

Li FP, Fraumeni JF Jr, Mulvihill JJ, Blattner WA, Dreyfus MG, Tucker MA and Miller RW (1988) A cancer family syndrome in twenty-four kindreds. *Cancer Res* **48**: 5358–5362

Li J, Yen C, Liaw D, Podsypanina K, Bose S, Wang SI, Puc J, Miliaresis C, Rodgers L, McCombie R, Bigner SH, Giovanella BC, Ittmann M, Tycko B, Hibshoosh H, Wigler MH and Parsons R (1997) *PTEN*, a putative protein tyrosine phosphatase gene mutated in human brain, breast and prostate cancer. *Science* **275**: 1943–1947

Malkin D, Li FP, Strong LC, Fraumeni JF Jr, Nelson CE, Kim DH, Kassel J, Gryka MA, Bischoff FR, Tainsky MA and Friend SH (1990) Germ line p53 mutations in a familial syndrome of breast cancer, sarcomas, and other neoplasms. *Science* **250**: 1233–1238

Myers MP, Stolarov JP, Eng C, Li J, Wang SI, Wigler MH, Parsons R and Tonks NK (1997) P-TEN, the tumor suppressor from human chromosome 10q23, is a dual-specificity phosphatase. *Proc Natl Acad Sci USA* **94**: 9052–9057

Okamoto A, Demetrick DJ, Spillare EA, Hagiwara K, Perwez Hussain S, Bennett WP, Forrester K, Gerwin B, Serrano M, Beach DH and Harris CC (1994) Mutations and altered expression of *p16$^{INK4}$* in human cancer. *Proc Natl Acad Sci USA* **91**: 11045–11049

Quelle DE, Zindy F, Ashmun RA and Sherr CJ (1995) Alternative reading frames of the *INK4a* tumor suppressor gene encode two unrelated proteins capable of inducing cell cycle arrest. *Cell* **83**: 993–1000

Steck PA, Pershouse MA, Jasser SA, Yung WKA, Lin H, Ligon AH, Langford LA, Baumgard ML, Hattier T, Davis T, Frye C, Hu R, Swedlund B, Teng DHF and Tavtigian SV (1997) Identification of a candidate tumour suppressor gene, *MMAC1*, at chromosome 10q23.3 that is mutated in multiple advanced cancers. *Nature Genet* **15**: 356–362

Tsou HC, Teng DH-F, Li Ping X, Brancolini V, Davis T, Hu R, Xun Xie X, Gruener AC, Schrager CA, Christiano AM, Eng C, Steck P, Ott J, Tavtigian SV and Peacocke M (1997) The role of *MMAC1* mutations in early-onset breast cancer: causative in association with Cowden syndrome and excluded in *BRCA1*-negative cases. *Am J Hum Genet* **61**: 1036–1043

Varley JM, Evans DGR and Birch JM (1997*a*) Li–Fraumeni syndrome: a molecular and clinical review. *Br J Cancer* **76**: 1–14

Varley JM, McGown G, Thorncroft M, Santibanez Koref MF, Kelsey AM, Tricker KJ, Evans DGR and Birch JM (1997*b*) Germline mutations of *TP53* in Li–Fraumeni families: an extended study of 39 families. *Cancer Res* **57**: 3245–3252

© Cancer Research Campaign 1999
